# Understanding the Role of Yes-Associated Protein (YAP) Signaling in the Transformation of Lens Epithelial Cells (EMT) and Fibrosis

**DOI:** 10.3390/biom13121767

**Published:** 2023-12-09

**Authors:** Aftab Taiyab, Yasmine Belahlou, Vanessa Wong, Saranya Pandi, Madhu Shekhar, Gowri Priya Chidambaranathan, Judith West-Mays

**Affiliations:** 1 Department of Pathology and Molecular Medicine, Faculty of Health Sciences, McMaster University, Hamilton, ON L8N 3Z5, Canada; belahloy@mcmaster.ca (Y.B.); wongv11@mcmaster.ca (V.W.); 2 Department of Immunology and Stem Cell Biology, Aravind Medical Research Foundation, Madurai 625020, Tamil Nadu, India; saranya.pandi@aravind.org (S.P.); gowri@aravind.org (G.P.C.); 3 Cataract and IOL Services, Aravind Eye Hospital and Post Graduate Institute of Ophthalmology, Madurai 625020, Tamil Nadu, India; madhushekhar93@gmail.com

**Keywords:** EMT, MMP9, TGF-β, lens, PCO, α-SMA, YAP, mechanotransduction

## Abstract

Fibrotic cataracts, posterior capsular opacification (PCO), and anterior subcapsular cataracts (ASC) are mainly attributed to the transforming growth factor-β (TGFβ)-induced epithelial-to-mesenchymal transition (EMT) of lens epithelial cells (LECs). Previous investigations from our laboratory have shown the novel role of non-canonical TGFβ signaling in the progression of EMT in LECs. In this study, we have identified YAP as a critical signaling molecule involved in lens fibrosis. The observed increase in nuclear YAP in capsules of human ASC patients points toward the involvement of YAP in lens fibrosis. In addition, the immunohistochemical (IHC) analyses on ocular sections from mice that overexpress TGFβ in the lens (TGFβ^tg^) showed a co-expression of YAP and α-SMA in the fibrotic plaques when compared to wild-type littermate lenses, which do not. The incubation of rat lens explants with verteporfin, a YAP inhibitor, prevented a TGFβ-induced fiber-like phenotype, α-SMA, and fibronectin expression, as well as delocalization of E-cadherin and β-catenin. Finally, LECs co-incubated with TGFβ and YAP inhibitor did not exhibit an induction in matrix metalloproteinase 2 compared to those LECs treated with TGFβ alone. In conclusion, these data demonstrate that YAP is required for TGFβ-mediated lens EMT and fibrosis.

## 1. Introduction

Cataracts remain the primary cause of blindness worldwide, affecting nearly 95 million people, with the incidence rising due to a global aging population [1,2,3,4]. Primary cataracts are further classified into non-fibrotic cataracts, comprising nuclear and cortical cataracts, and fibrotic cataracts, encompassing anterior sub-capsular cataracts (ASC). Prior research from various laboratories, including ours, has established that the epithelial-to-mesenchymal transition (EMT) of lens epithelial cells (LECs) is the primary cause of fibrosis of the ocular lens or fibrotic cataract that ultimately results in opacification of the lens and vision loss [5,6,7,8]. While surgical replacement of the cataractous lens with an artificial intra-ocular lens (IOL) is the most effective and widely practiced treatment for cataracts, post-operative secondary complications such as secondary cataracts or posterior capsular opacification (PCO) may still arise in ~14–18% of patients undergoing cataract surgery [9]. Similar to ASC, PCO also involves the EMT of LECs. While most of the anterior lens tissue is removed during the cataract surgery, the equatorial LECs and the posterior capsule remain intact. Cataract surgery injury results in mechanical perturbation of LECs and induces a wound-healing response where the LECs start to migrate to the posterior side of the capsular bag. The migrating LECs also become exposed to otherwise inaccessible aqueous humor containing cytokines such as transforming growth factor beta (TGFβ) that are known to promote EMT. EMT results in cellular and molecular changes in LECs, including enhanced capabilities of migration and production and deposition of extracellular matrix (ECM) components, leading to posterior capsular wrinkling [10,11,12]. The transformed LECs are marked by decreased expression of epithelial cadherin (E-cadherin), an important surface protein that maintains cell-to-cell adhesion, and increased expression of proteins that facilitate cell migration, such as alpha-smooth muscle actin (α-SMA) and vimentin, all of which are known EMT indicators [11,13,14,15].

TGFβ is identified as the major EMT-inducing cytokine known to promote transdifferentiation of LECs to mesenchymal cells. Specifically, a positive correlation exists between the increased concentration of TGFβ following cataract surgery in the aqueous humor and lens fibrotic disorders, including ASC and PCO [5,16,17]. Several experimental rodent model systems, including transgenic mice overexpressing TGFβ in the lens and adenoviral-mediated gene transfer of TGFβ to the rodent lens, have been established to understand the role of TGFβ in lens fibrosis. The lenses in both models showed α-SMA-overexpressing lenticular plaque and EMT of LECs indicative of ASC formation, resembling what is observed in humans [8,18,19]. Furthermore, incubation of whole rat lenses in active TGFβ also resulted in plaque formation/opacification due to EMT of LECs [18,19]. In addition, incubation of ex vivo rats and mouse lens epithelial explants with active TGFβ generated spindle-shaped myofibroblasts that exhibited decreased E-cadherin, increased expression of α-SMA, and increased accumulation of ECM [20,21,22].

Tissue injury, including lens tissue damage upon cataract surgery, causes changes in cellular and mechanical cues that lead to activation of mechanotransduction signaling. Yes-associated protein (YAP), the core downstream transcription co-activator of the Hippo signaling pathway, is the key mediator of mechanotransduction signaling that works in a complex with another transcriptional co-activator with PDZ-binding motif (TAZ) following tissue injury [23,24]. The nuclear YAP/TAZ complex binds to and activates the terminal regulator TEA domain (TEAD), a transcription factor known to regulate genes involved in cell proliferation and EMT [25,26,27]. Furthermore, the nuclear interaction between YAP/TAZ and Smad 2/3 has been shown to promote TGFβ-induced EMT in keratinocytes, thereby showing a link between YAP, TGFβ, and EMT [28]. The stretching of whole lenses has been linked to YAP-mediated increased proliferation of LECs, a phenomenon that may contribute to age-related lens pathologies, including presbyopia and cataracts [29]. In addition, a recent report linked the stretching of LECs upon actin reorganization to nuclear translocation of YAP and EMT, suggesting a potential role of YAP during EMT in the lens [30]. Despite the previous findings, the direct correlation between YAP and TGFβ-induced EMT in LECs and, thus, lens fibrosis has yet to be established. In the current study, we show the involvement of YAP signaling in lens fibrosis and provide evidence for the requirement of YAP in TGFβ-induced lens EMT.

## 2. Materials and Methods

### 2.1. Collection and Immunostaining of Anterior Lens Capsule from Anterior Subcapsular Cataract (ASCC) Patients

Anterior lens capsules were collected from anterior subcapsular cataract patients (*n* = 3; age (mean ± SD): 55.3 ± 7.57 years) during cataract surgery at Aravind Eye Hospital and Postgraduate Institute of Ophthalmology, Madurai. Samples were preserved in minimal essential medium with 20% fetal bovine serum and placed on ice immediately after anterior continuous curvilinear capsulorhexis. Informed consent was obtained from the patients, and this study was approved by the Institutional Review Board of Aravind Medical Research Foundation (IRB2018015BAS). This study adhered to the tenets of the Declaration of Helsinki.

The central 6 mm anterior lens capsule along with epithelium was spread on a glass slide, fixed with 4% paraformaldehyde for 15 min, and was blocked with avidin biotin blocking system. After overnight incubation at 22 °C with the primary antibody α-SMA (Sigma, St. Louis, MO, USA, A5228), the corresponding biotinylated goat anti-mouse secondary antibody (1:200 dilution, Dako, Glostrup, Denmark, E0433) was added and incubated for 1 h at 22 °C. Streptavidin fluorescein isothiocyanate (FITC, BD Pharmingen, San Diego, CA, USA) was added at a dilution of 1:1000 (in 1x PBS) and incubated for 1 h at 22 °C in dark to visualize the marker. For double immunostaining, the sample was incubated overnight at 22 °C with anti-YAP (the second primary antibody, Cell Signaling Technology, Danvers, MA, USA, 52420), followed by the corresponding biotinylated anti-rabbit secondary antibody (1:200 dilution, Santacruz, Santa Cruz, MA, USA, SC-2491) and streptavidin-Alexa 633 (Thermofisher Scientific, Waltham, MA, USA) at a dilution of 1:1000 in 1× PBS. Between steps, the slides were washed thrice in 1× PBS, and the stained lens capsules were mounted with Vectashield mounting medium (Burlingame, CA, USA) containing 4,6-diamidino-2-phenylindole (DAPI). During the immunostaining, samples without the addition of primary antibody were used as a negative control.

Acquisition of the immunostained capsule was carried out using a laser scanning microscope (Leica SP8 confocal microscope, Leica, Wetzlar, Germany). Briefly, fluorescent Z stack images were acquired using the following settings: the emission bandwidth for DAPI ranged from 358 to 441 nm; FITC ranged from 496 to 535 nm using laser blue 488, and Alexa Fluor ranged from 633 from 640 to 725 nm using laser red 633 nm. Using the above-mentioned parameters, the images were acquired at 40× objective.

### 2.2. TGFβ Transgenic Mice (TGFβ^tg^)

All animal experiments adhered to the animal research guidelines of association of research in vision and ophthalmology (ARVO) and were approved by animal research ethics committee at McMaster University prior to the experiments. TGFβ1 transgenic mice were obtained from Dr. Paul Overbeek (Baylor College of Medicine) and contain a porcine TGFβ1 cDNA construct with an αA-crystallin promoter designed for lens-specific expression of active TGF-β1 on a FVB/N/C57BL/6J genetic background [31]. The TGFβ1 transgene was identified using primers specific for the simian virus 40 sequences in the transgene. The sense primer (5′-GTGAAGGAACCTTACTTCTGTGGTG-3′) and the antisense primer (5′-GTCCTTGGGGTCTTCTACCTTTCTC-3′) yield a 300-bp fragment. PCR reactions were performed for 36 cycles using the following conditions: initial heating for 3 min at 94 °C, denaturation for 30 s at 94 °C, annealing for 1 min at 57 °C, and extension for 1 min at 72 °C. A final extension was performed for 10 min at 72 °C. Agarose gel electrophoresis (1.5% agarose) with ethidium bromide detection was used to visualize the PCR reaction products.

### 2.3. Hematoxylin and Eosin (H&E Staining) and Immunohistochemistry (IHC)

The eyes from 3 females and 2 males of postnatal (P) day 30 TGFβ^tg^ and equal number of wild-type littermates were extracted from euthanized mice and fixed in 4% formaldehyde (PFA) for 24 h at 4 °C for staining and immunohistochemistry. Following fixation, eyes were preserved in 70% ethanol at 4 °C for paraffin sections. The eyes were sent for processing and embedded in paraffin wax (Paraplast tissue embedding media, Fisher Scientific, Waltham, MA, USA). The paraffin blocks were subsequently sectioned into 4 μm thick sections for hematoxylin and eosin (H&E) or immunohistochemical staining. The paraffin embedded sections were deparaffinized in xylene and rehydrated in 100%, 95%, and 70% ethanol. The sections were then distilled in water and stained for H&E. For IHC, the distilled sections were treated with 10 mM of sodium citrate (pH 6.0) for antigen retrieval. The sections were blocked with 5% normal serum diluted with 1× PBS from the host animal of the secondary antibody for 1 h. After the blocking step, sections were incubated in appropriate primary antibodies with 1× PBS solution mixture overnight at 4 °C. Primary antibodies include a-SMA-FITC (1:100, Sigma, F3777) and YAP (1:200, cell signaling, 4912S). The next day, the sections were incubated with AlexaFluor secondary antibodies (1:200, Invitrogen, Molecular Probes, Burlington, ON, Canada) in addition to 1.5% normal serum diluted in 1× PBS to detect the primary antibodies. The sections were incubated for 1 h, which was followed by 3 × 0.1% Tween-20 solution in 1× PBS. The slides were then mounted using the ProLong Gold with the nuclear stain DAPI (4′,6-diamidino-2-phenylindole) (Thermofisher, Waltham, MA, USA) and imaged with a Leica DM6 B microscope and acquired using LasX imaging software 1.4.5.

### 2.4. Culturing Rat Lens Epithelial Explants

All animal experiments adhered to the animal research guidelines of association of research in vision and ophthalmology (ARVO) and were approved by animal research ethics committee at McMaster University prior to the experiments. Rat lens epithelium was explanted on their native capsular bag to create an ex vivo LEC model (rat LEC) for observing EMT. This was carried out by extracting eyes from 17–19-day-old Wister rats after euthanizing them via CO_2_ overdose and cervical dislocation. The eyes were then placed in M199 media (Gibco by Life Technologies, Carlsbad, CA, USA) containing gentamycin (10 mg/mL, Gibco by Life Technologies, USA), pen strep (Invitrogen, Waltham, MA, USA), and fungizone (Invitrogen, USA) at 37 °C. The lens was then extracted by tearing the eye open from the back (right by the optic nerve) using two teethed forceps under a dissecting microscope (Leica MZ16, Wetzlar, Germany). All other eye structures were discarded. The collected lenses were then transferred into fresh plates of media (35 mm). Two sharp forceps were used to make an incision on the posterior end of the lens to allow for peeling of the capsular bag. After the capsular bag was removed, it was pinned to the plate using a blunt tool. These explants were then incubated at 37 °C in an incubator (5% CO_2_, and 95% humidity).

### 2.5. Treating LECs with TGF-β and Inhibitors

After 24 h of explanting, selected explants were treated with 2 mL of media containing 100 nM of Verteporfin (in <0.5% of DMSO (Sigma-Aldrich Corp, St. Louis, MO, USA)), a YAP inhibitor, and incubated at 37 °C for 1 h. Few plates were also co-treated with 6 ng/mL of recombinant human TGFβ2 in <0.5% of DMSO (R&D Systems, Minneapolis, MN, USA) and incubated for 48 h. Vehicle (DMSO) treated LECs were considered as untreated/control LECs.

### 2.6. Immunofluorescence Staining

After 48 h, lens explants were fixed in 4% paraformaldehyde (PFA) at room temperature for 10 min (on a shaker at low speed). The PFA was then discarded, and the plates were washed with 1× phosphate-buffered saline solution three times (1× PBS; Invitrogen, USA). Every explant was then lifted from its respective plate surface with the same blunt tool used to pin them down and placed into separate culture tubes labeled by treatment type along with PBS. The PBS was then aspirated out of the tubes containing explants, and 5% normal donkey serum (NDS; Invitrogen, USA) in permeabilizing buffer (0.1% Triton-X-100 and 0.5% sodium dodecyl sulfate (SDS) in PBS) was used to block and permeabilize the explants. The tubes were covered and rocked gently at room temperature (on a shaker) for 1 h. Next, explants were treated with primary antibody at a 1:200 dilution and incubated overnight at 4 °C. The antibodies used were for β-catenin (mouse anti-β-catenin; BD Transduction, San Diego, USA: 610153), fibronectin (rabbit anti-fibronectin, Millipore, Burlington, MA, USA: AB1954), and YAP (rabbit anti-YAP, Cell Signaling Technology, USA: 4912S). After 24 h, the explants were washed with 1× PBS 3 times for 10 min each (by gentle rocking). The secondary antibody was then added to the explants at a 1:200 dilution and covered and incubated at room temperature for 1 h under gentle rocking. Donkey anti-mouse antibody conjugated to Alexa Fluor^®^488 (Invitrogen, USA) for Β-catenin and donkey anti-rabbit for fibronectin and YAP (Invitrogen, USA) were the secondary antibodies used in this step. Explants were then washed 3 more times with 1× PBS (as was carried out previously) for 10 min each. Later, the stained explants were mounted onto slides (up to 3 explants per glass slide) using the Prolong Gold antifade reagent with 4′-6-diamidino-2-phenylindole (DAPI, Invitrogen by Life Technologies). DAPI was used to help visualize the nucleus. Finally, fluorescent staining was visualized using a Leica fluorescent microscope (DM6 B, Leica, Wetzlar, Germany), and images were captured using a Leica CTR6 LED camera. Image J was then used to determine total mean fluorescence (for the fibronectin stain) and nuclear fluorescence (for the YAP stain). Statistical analysis of these data were performed using GraphPad Prism 6 (ANOVA).

### 2.7. Reverse Transcriptase (RT) PCR

Following the treatments, the lens explants were washed with ice-cold 0.1% diethyl pyro carbonate phosphate-buffered saline (DEPC-PBS, Invitrogen, BioShop^®^, Burlington, ON, Canada). Explants were then removed from their respective plates using a blunt tool. RNA was then isolated from explant samples using the RNeasy^®^ kit (QIAGEN, Germantown, MD, USA) in preparation for subsequent reverse transcription as directed by the manufacturer’s protocol. Following the isolation, the RNA was estimated using NanoDrop (Thermo Fisher Scientific 2000 spectrophotometer, Waltham, MA, USA). The purity of the RNA was also determined by performing agarose gel electrophoresis. The RNA samples were then subjected to reverse transcription (RT) using the High-Capacity cDNA Reverse Transcription Kit (Applied Biosystems, Waltham, MA, USA) to make cDNA using the manufacturer’s instructions. The cDNA was then subjected to PCR for the MMP2, αvβ6 and GAPDH using the following primers: MMP2: F-5′-GGCCGTACAATCTTCACTGCA–3′, R-5′-AGCACCTTTCTTTGGGCACAA-3′; MMP9:F-5′-TCATTCTTCAGTGCCGGAAGC-3′, R-5′-GGACACATAGTGGGAGGAGCT-3′; GAPDH: F-5′-GTATTGGGCGCCTGGTTATC-3′, R-5′-CGCTCCTGGAAGATGGTGATGG-3′. The product was then electrophoresed using 2% agarose gel. Next, the gel was imaged using the VWR International Co. Syngene G:Box (VWR, Burlington, Canada), and the bands were processed using the G:Box Chemi XRQ GeneSys version 3 (Canada) software. Finally, Image J was used to determine relative band fluorescence for fold change calculations, and GraphPad was used to perform an ANOVA statistical analysis.

## 3. Results

### 3.1. Human Fibrotic Lenses Show Nuclear Expression of YAP

**Figure 1:**
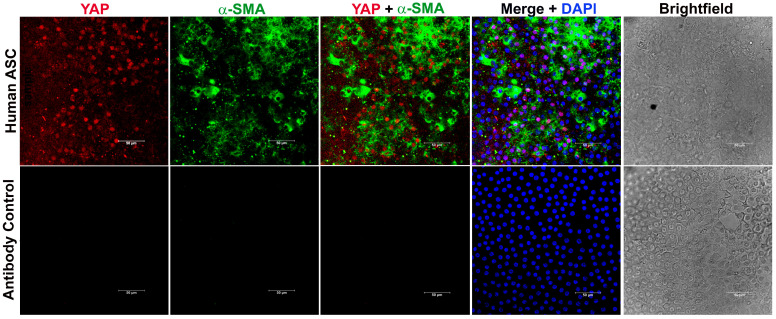
Anterior lens capsules collected from human anterior subcapsular cataract (ASC) patients were stained for YAP and α-SMA and mounted with vectashield mounting medium containing DAPI. During immunostaining, samples without addition of primary antibody were used as negative control (panel 2). Image acquisition of the immuno-stained capsules was conducted using a laser scanning microscope (Leica SP8 confocal microscope, Leica, Wetzlar, Germany) using 40× objective. Scale bars, 50 µm.

YAP signaling is known to be involved in the induction of fibrosis of various tissues, including the heart [32], lung [33], and kidney [32]. However, the direct involvement of YAP in lens fibrosis is not completely understood. To investigate the relevance of YAP in lens fibrosis, we collected samples of lens capsules harboring LECs from human patients who have undergone anterior subcapsular cataract (ASC) surgery and probed for the presence of YAP using immunohistochemistry (IHC). Confocal analyses of the immunostained fibrotic lens capsules showed the presence of cytoplasmic YAP, along with α-SMA, a known EMT marker (Figure 1). In addition, the presence of YAP in the nucleus was also observed in the LECs expressing α-SMA from the ASC lens capsule (Figure 1). However, there were LECs that did not express YAP but were noted to be positive for α-SMA, thereby revealing a population of LECs that were either positive for α-SMA or YAP alone and other populations of LECs that were positive for both α-SMA and YAP (Figure 1). As expected, YAP and α-SMA were absent from lens capsules stained for secondary antibody alone (Figure 1; lower panel). These IHC data indicate a positive correlation between the presence of YAP and lens fibrosis and sets the premise for further investigating the role of YAP in lens fibrosis.

### 3.2. Upregulation of Active TGFβ in Mouse Lenses Results in Increased YAP Expression in the Fibrotic Plaques

The role of TGFβ in fibrosis has been well established [34], and overexpression of active TGFβ in mouse lenses (TGFβ^tg^) is known to form anterior lenticular fibrotic plaques that are known to overexpress α-SMA [31]. The interplay between TGFβ and YAP signaling during wound healing in other model systems, such as skin, has been well documented [35]. However, the direct influence of TGFβ signaling on YAP expression in the lens is not known.

**Figure 2:**
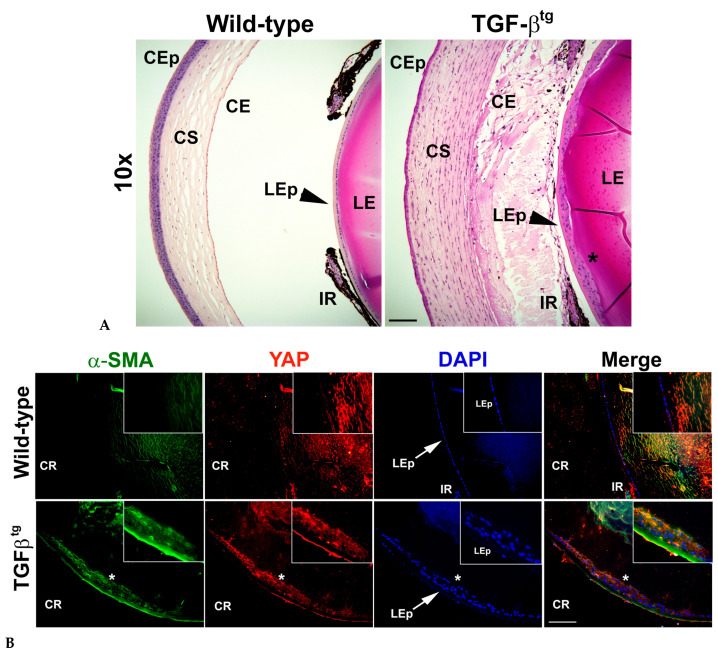
Overexpression of TGFβ in the mouse lens results in lenticular plaque formation and increase in expression of yes-associated protein (YAP) and alpha-smooth muscle actin (α-SMA) in the lens epithelium. (**A**) H&E staining of wild-type ocular section shows a single layer of epithelial cells (LEp) (arrowheads). Overexpression of TGFβ in the lens (TGFβ^tg^) leads to increased cell proliferation, resulting in plaque formation (*) in the anterior lens. Images were acquired using the 20× lens of Leica DM6 fluorescence microscope. CEp—corneal epithelium; CS—corneal stroma; CE—corneal endothelium; IR—iris; LE—lens; LEp—lens epithelium. (**B**) Immunohistochemistry (IHC) for YAP and alpha-smooth muscle actin (α-SMA) was performed using ocular sections (similar to that used in (**A**)) from TGFβ^tg^ mice and their wild-type littermates (*n* = 5 eyes from different animals). The lens epithelium (LEp) of the wild-type eyes exhibits a monolayer of cells (arrow in panel 1). The mutant lenses show multilayered cells (ingrowth of lens epithelial cells; arrow in panel 2) that overexpress both YAP and α-SMA, resembling fibrotic plaques (*). Nuclei were counter-stained with DAPI. Images were acquired using the 20× lens of Leica DM6 fluorescence microscope. Inset images were acquired using 63× lens of Leica DM6 fluorescence microscope. CR—cornea for orientation purposes. Scale bars are set to 100 µm for all images.

To demonstrate the effect of active TGFβ signaling on YAP expression, we used lenses from TGFβ^tg^ mouse and their wild-type littermates. The hematoxylin and eosin (H&E) staining in Figure 2A (panel 2; *n* = 4) confirmed the formation of lenticular plaques in TGFβ^tg^ lenses when compared to the lenses from wild-type mice (Figure 2A, panel 1; *n* = 4). Interestingly, IHC analyses on TGFβ^tg^ sections showed an increase in YAP expression along with an upregulation of α-SMA, specifically in the fibrotic plaques of the lens (Figure 2B). At the same acquisition intensity, both YAP and α-SMA were not expressed in the LECs of wild-type mouse lenses (Figure 2B, panel 1; *n* = 6), suggesting that upregulation of YAP and α-SMA was unique to overexpression of TGFβ in the lens.

### 3.3. Inhibition of YAP Signaling Prevents TGF-β Induced Fibrotic Phenotype of LECs, Upregulation of α-SMA and YAP, and Nuclear Translocation of YAP

**Figure 3:**
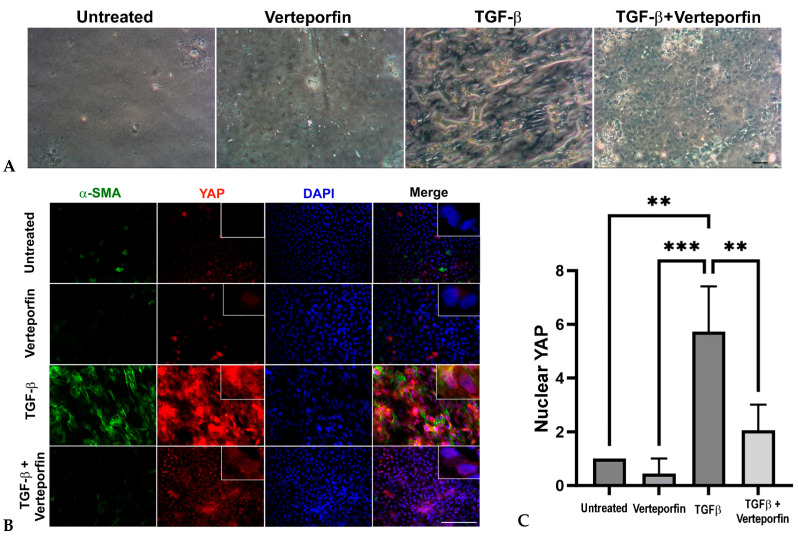
Inhibition of YAP by verteporfin prevents changes in LEC morphology, prevents decreases in TGFβ-induced α-SMA and YAP expression, and blocks nuclear translocation of YAP. (**A**) Rat lens explants were treated with TGFβ (6 ng/mL) in the presence or absence of 100 nM verteporfin for 48 h (*n* = 6 explants per treatment). Verteporfin prevented TGFβ-induced fiber-like phenotype of LECs. The phase contrast images were acquired using 10× brightfield objective lens of Leica DM IL LED microscope using the Moticam BTU camera attachment. Scale bars are set to 100 µm. (**B**) Untreated (vehicle-treated) rat LECs and rat LECs incubated with TGFβ for 48 h in the presence or absence of verteporfin (*n* = 6 explants per treatment) were fixed and stained for YAP (red) and α-SMA (green) and mounted onto glass slides using the prolong gold antifade reagent containing DAPI (blue). Pretreatment of rat LECs with verteporfin prevented TGFβ-induced nuclear translocation of YAP and α-SMA overexpression. Images were obtained using the 40× lens of Leica DM6 fluorescence microscope. Scale bars are set to 100 µm. (**C**) Graph showing statistical analysis (ANOVA–Tukey multiple comparison test) of YAP nuclear fluorescence fold change. An increase in nuclear YAP was observed in LECs stimulated with TGFβ when compared to untreated LECs (**, *p* < 0.01) or LECs treated with verteporfin alone (***, *p* < 0.001). A significant decrease in TGFβ-induced nuclear YAP was observed in LECs pretreated with verteporfin (**, *p* < 0.01 TGFβ-treated vs. TGFβ + verteporfin-treated LECs). Error bars indicate standard deviation.

To determine if YAP is critical during lens fibrosis, we employed verteporfin in combination with TGFβ to inhibit YAP in rat lens explants (rat LECs). Verteporfin is a known inhibitor of YAP signaling that induces sequestration of YAP in the cytoplasm by increasing the levels of 14-3-3σ, a YAP chaperone protein that retains YAP in the cytoplasm and targets it for proteasome-mediated degradation [36], thereby disrupting YAP-TEAD-mediated downstream transcription [37]. We standardized 100 nM to be the effective concentration of verteporfin, a concentration at which the inhibitor was not toxic to the cells but was able to inhibit morphological changes induced by TGFβ stimulation. The untreated and verteporfin-treated rat LECs showed a continuous layer of cells (Figure 3A) that appeared to be arranged in a cobblestone manner, a morphological phenotype of normal lens epithelial cells. Upon stimulation with TGFβ, the cellular morphology of rat LECs appeared spindle-like, and the capsule began to wrinkle (Figure 3A). The pretreatment of rat LECs with verteporfin prevented TGFβ-induced changes, including capsular wrinkling and spindle-like morphology of LECs (Figure 3A). The rat LECs were further probed to investigate the status of YAP and α-SMA, a known EMT marker. Immunofluorescence analyses revealed an increase in α-SMA and YAP expression in TGFβ-treated rat LECs (Figure 3B). In contrast, incubation of LECs with verteporfin prior to stimulation with TGFβ resulted in inhibition of both α-SMA and YAP expression when compared to rat LECs treated with TGFβ alone. The statistical analyses of immunofluorescence images revealed a ~5.7-fold and a ~12-fold increase in nuclear accumulation of YAP in TGFβ-treated LECs when compared to the untreated rat LECs (*p* < 0.01; Figure 3C) and LECs incubated with verteporfin alone (*p* < 0.001; Figure 3C), respectively. In contrast, the LECs treated with both TGFβ and verteporfin showed a ~3-fold reduction in nuclear YAP when compared to LECs incubated with TGFβ alone (*p* < 0.01; Figure 3C). We did not observe a significant accumulation of YAP in the nucleus between untreated/verteporfin-treated LECs and LECs treated with both TGFβ and verteporfin (Figure 3C). In addition, incubation of LECs with verteporfin was not able to prevent TGFβ-induced nuclear translocation of TAZ, an important co-factor of YAP, thereby showing the specificity of verteporfin towards YAP inhibition (Figure S1). These results suggest that inhibition of YAP by verteporfin prevents TGFβ-induced nuclear accumulation of YAP.

### 3.4. Inhibition of YAP Signaling Prevents TGFβ-Induced Delocalization of E-Cadherin and β-Catenin

**Figure 4:**
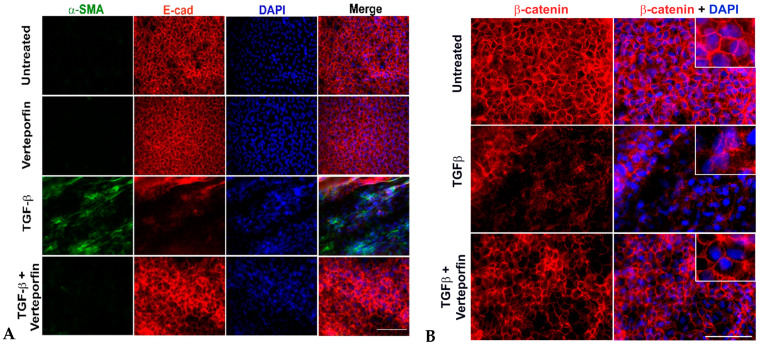
Inhibition of YAP blocks EMT. Rat lens epithelial explants (LECs) were treated with TGFβ (6 ng/mL) in the presence or absence of verteporfin (100 nM) (*n* = 9 explants per treatment group) for 48 h. Paraformaldehyde (PFA) fixed explants were stained for either (**A**) α-SMA and E-cadherin (E-cad) or (**B**) β-catenin and mounted using prolong gold antifade reagent containing DAPI as a nuclear stain. (**A**) Incubation of LECs with TGFβ results in α-SMA expression and E-cadherin degradation (panel 3). Verteporfin was able to prevent TGFβ-induced α-SMA expression, and E-cadherin delocalization and degradation (panel 4). Images were acquired using the 40× lens of Leica DM6 fluorescence microscope. (**B**) The untreated (vehicle-treated) explants showed membrane localization of β-catenin. Delocalization of β-catenin upon TGFβ treatment was prevented by verteporfin. Images were acquired using the 63× oil objective lens of Leica DM6 fluorescence microscope. Scale bars are set to 100 µm.

The nuclear interaction between YAP/TAZ and Smad 2/3 has been shown to promote TGFβ-induced EMT in keratinocytes, thereby showing a link between YAP, TGFβ, and EMT [28]. In addition, a recent report linked the stretching of LECs upon actin reorganization to nuclear translocation of YAP and EMT, suggesting a potential role of YAP during EMT in the lens [30]. To establish the direct link between TGFβ, YAP, and EMT in the lens, we investigated the expression of key EMT markers, α-SMA, and E-cadherin. As expected, the untreated and verteporfin alone-treated LECs showed no expression of α-SMA and peripheral localization for the epithelial marker, E-cadherin (Figure 4A). In contrast, TGFβ treatment led to an increase in α-SMA expression and resulted in depletion of E-cadherin, leading to a loss of peripheral staining of E-cadherin. Noticeably, treating LECs with TGFβ in the presence of verteporfin prevented TGFβ-induced E-cadherin degradation and α-SMA expression. The staining profile for α-SMA and E-cadherin in LECs incubated with TGFβ and verteporfin resembled that of untreated or verteporfin-alone-treated LECs.

The disruption of E-cadherin is believed to activate β-catenin, another key component of the cell junction that is known to regulate the expression of key EMT-associated proteins, including fascin, αSMA, and MMP9 during TGFβ-induced EMT in the lens [38]. The untreated LECs showed membranous localization of β-catenin that was disrupted in the presence of TGFβ (Figure 4B). The pretreatment of LECs with verteporfin prevented the loss of peripheral localization of β-catenin (Figure 4B), indicating a key role of YAP in the maintenance of both E-cadherin and β-catenin.

### 3.5. Inhibition of YAP Signaling Blocks TGFβ-Induced Fibronectin Expression

**Figure 5:**
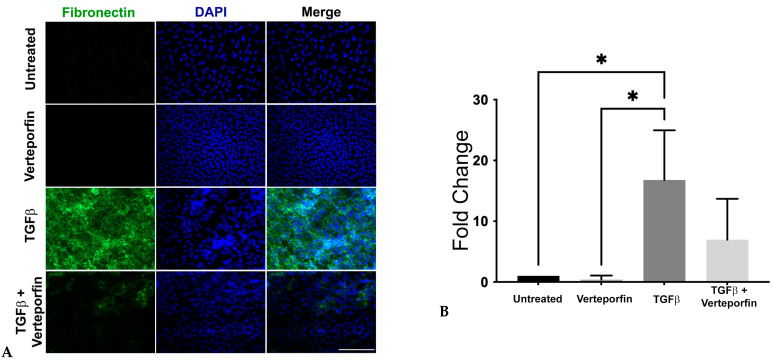
Expression and localization of fibronectin. Rat LECs treated with TGFβ alone (6 ng/mL) or along with verteporfin (100 nM) were incubated for 48 h (*n* = 9 explants per treatment) and then fixed in PFA followed by staining for fibronectin (green). (**A**) Incubation of rat LECs with TGFβ results in upregulation of fibronectin. Images were obtained using the 40× lens of Leica DM6 fluorescence microscope with scale bars set to 100 µm. (**B**) Graph showing total mean fluorescence fold change in fibronectin expression (ANOVA–Tukey multiple comparison test) (*, *p* < 0.05 for untreated (vehicle-treated) or verteporfin-treated vs. TGFβ). Error bars indicate standard deviation.

Another crucial molecule involved in myofibroblast differentiation during TGFβ-induced EMT is fibronectin. It plays a significant role in the activation of TGFβ and the conversion of mechanical cues into biochemical cues in the lens upon surgery [39,40]. In tumorigenic epithelial cells, YAP regulates the expression of TGFβ-responsive genes, including fibronectin and collagens, through its association with the Smad signaling pathway [41]. Therefore, we were interested in investigating the status of fibronectin in TGFβ-stimulated LECs upon inhibition of YAP signaling. Immunofluorescence analyses revealed the absence of fibronectin in the untreated and verteporfin-alone treated LECs. However, upon stimulation by TGFβ, the LECs showed a significant increase in the presence of fibronectin. In contrast, inhibition of YAP signaling prevented TGFβ-induced fibronectin expression (Figure 5A). Further statistical analyses of the immunofluorescence images revealed a ~17-fold increase in fibronectin expression in LECs treated with TGFβ when compared to untreated or verteporfin-treated LECs (*p* < 0.05; Figure 5B). In contrast, verteporfin pretreated LECs followed by stimulation with TGFβ LECs showed a ~3-fold decrease in fibronectin when compared to LECs incubated with TGFβ-alone (Figure 5B).

### 3.6. Inhibition of YAP Downregulates TGFβ-Induced MMP2 Expression

Matrix remodeling is an important requisite for EMT, and matrix metalloproteinases (MMPs) play a key role in matrix modification during the transformation of epithelial cells. Specifically, MMP2 and MMP9 are known to be important during lens EMT, and inhibition or absence of MMP2, MMP9, or both have been correlated with inhibition of TGFβ-induced EMT in the lens [9,21,42,43,44]. Considering the importance of MMP2 and MMP9 during TGFβ-mediated lens EMT, we investigated the role of YAP signaling in the regulation of MMP2 and MMP9 expression in LECs stimulated with TGFβ using reverse transcriptase (RT)-PCR. We observed a significant increase in MMP2 expression in LECs incubated with TGFβ (~5-fold) when compared to untreated (*n* = 3, *p* < 0.0001) or verteporfin-treated LECs (*n* = 3, *p* < 0.0001). Interestingly, verteporfin pretreated LECs incubated with TGFβ showed a significant reduction in MMP2 expression (~3.5-fold) when compared to LECs incubated with TGFβ alone (*n* = 3, *p* < 0.001). Surprisingly, we did not observe a significant change in MMP9 expression in TGFβ-treated LECs preincubated with verteporfin when compared to LECs stimulated with TGFβ alone (Figure S2). These findings suggest that MMP2 expression is reliant on YAP signaling during TGF-β induced EMT in the lens.

## 4. Discussion

Understanding the causative mechanisms that drive EMT leading to the development of fibroproliferative diseases, including ASC and PCO, is necessary if we hope to find a cure for these pathological conditions. We, and others, have previously shown the role of TGFβ influenced pathways, including β-catenin and Rho signaling pathway, in regulating EMT-driven lens fibrosis that is marked by actin reorganization [38,44,45,46]. In humans, in addition to increased TGFβ signaling, stretching of the lens resulted in nuclear YAP-induced LEC proliferation, which has also been associated with age-related cataracts [29]. Furthermore, a positive correlation has been established between Hippo/YAP signaling and fibroblast growth factor 2 (Fgf2)-induced LEC proliferation and its differentiation to fiber cells [47]. A recent investigation by Maddala and colleagues has linked actin reorganization in LECs during lens stretching to nuclear translocation of YAP, leading to EMT [30]. Our new IHC data using lenticular capsules isolated from human patients who have undergone ASC surgery revealed nuclear YAP along with an increase in α-SMA, indicating an involvement of YAP signaling in lens fibrosis (Figure 1). The ASC capsule showed a variable pattern of staining for α-SMA, with the highest expression being at the center of the capsule, as has been observed previously [48]. Indeed, we observed some α-SMA positive LECs that did not express YAP, thereby showing that a mixed population of LECs exists in human ASC capsules, one that is positive for both α-SMA and YAP and the other population that is positive for either α-SMA or YAP. The increase in YAP and α-SMA expression in the lenticular plaques of TGFβ^tg^ lenses, when compared to control lenses, establishes a link between TGFβ, YAP, and lens fibrosis (Figure 2). These data are of particular importance as increased active TGFβ in the aqueous humor is linked to the formation of cataracts in humans [17], thereby increasing the possibility of a key role of TGFβ-induced YAP signaling in lens fibrosis leading to the development of cataracts in humans. In addition, it is known that TGFβ suppresses the Hippo pathway in cancer cells [49], and the inactive Hippo pathway results in the activation of YAP signaling leading to EMT [50]. This link further ameliorates the possibility of the involvement of YAP signaling in lens EMT and fibrosis.

Using a previously established LEC explant model system [5,7,9], we found that incubation of rat LECs with active TGFβ leads to a fiber-like morphology that was prevented upon inhibition of YAP signaling by verteporfin. The prevention of TGFβ-induced morphological changes by verteporfin can be attributed to the inhibition of TGFβ-induced α-SMA expression, a contractile EMT marker, and a key contributor of morphological changes of epithelial cells to myofibroblasts during EMT [51,52]. In addition to α-SMA, our immunofluorescence analyses also revealed prevention of TGFβ-induced nuclear translocation of YAP in LECs co-treated with verteporfin, indicating a role of YAP in the regulation of α-SMA expression TGFβ-induced EMT in the lens (Figure 3). Our results are in conjunction with previous observations that have linked the presence of nuclear YAP to actin reorganization and EMT [29,30]. Interestingly, TAZ, a partner of YAP, did not show a decrease in TGFβ-induced nuclear translocation upon inhibition of YAP by verteporfin, thus showing the specificity of verteporfin to inhibit YAP and that TAZ alone is insufficient to induce EMT in LECs upon stimulation by TGFβ (Figure S1). Another known indicator of EMT is E-cadherin, a member of the adherens junction complex, the expression and peripheral membrane localization of which decreases in the presence of TGFβ in LECs [5,38,45]. The ability of verteporfin to prevent TGFβ-induced loss of E-cadherin and α-SMA overexpression clearly shows the involvement of YAP signaling during TGFβ-induced EMT of LECs (Figure 4). Due to the disruption of E-cadherin, the proteins of the adherens junction complex, including p120-catenin, β-catenin, and α-catenin also dislodges from the membrane, leading to destabilization of the adherens complex. This change in the adherens junction complex leads to the activation of β-catenin, which has been shown to play a key role in TGFβ-induced EMT in the lens through its association with Smad transcription factors, the core downstream transcription factor involved during TGFβ signaling [38,46,53]. The protection of loss of peripheral β-catenin by verteporfin in LECs stimulated with TGFβ suggests the ability of YAP to modulate the localization of β-catenin, and thus the activity of β-catenin by preventing the loss of adherens junction (Figure 4). Previously, we have shown that the interaction between β-catenin and Smad is a key event during TGFβ-induced EMT in the lens [46]. In processes such as self-renewal of human epithelial stem cells, YAP/TAZ plays a key role in the modulation of TGFβ signaling through their interaction with Smads and regulation of nucleocytoplasmic shuttling of Smad2/4 and Smad3/4 complexes [54]. Another mechanism through which YAP regulates TGFβ signaling is through its association with Smad7 at the TGFβ type 1 receptor, which results in subsequent ubiquitination and degradation of the receptor complex [55]. It is possible that in the lens, YAP might be acting either directly through its association with Smads (Smad2/3/4/7) or indirectly through its interaction with Smad/β-catenin or both to regulate TGFβ-induced EMT. However, further investigation is required to tease out the downstream molecules of TGFβ signaling that are influenced by YAP during TGFβ-induced fibrosis in LECs.

While active TGFβ is known to be the major inducer of lens EMT and fibrosis, studies conducted in lens injury model systems show the requirement of upstream events for sustained TGFβ activation [56]. The downstream events arising from active TGFβ include upregulation of TGFβ and extracellular matrix (ECM) proteins such as fibronectin that interacts and stores inactive latent form of TGFβ in a complex with latent TGFβ binding protein 1 (LTBP1) and latent binding peptide (LAP) [39,40]. The link between fibronectin and TGFβ activation has been established by studies conducted on injured lenses from fibronectin conditional knockout mice that failed to activate TGFβ downstream signaling and showed a delayed fibrotic response when compared to injured lenses from wild-type mice [40]. Consistent with previous studies, we observed a significant upregulation of fibronectin expression in LECs stimulated with TGFβ. The ability of verteporfin to block TGFβ-induced upregulation of fibronectin in LECs demonstrates the role of YAP in the modulation of fibronectin expression and, thus, activation of TGFβ during TGFβ-mediated EMT in the lens (Figure 5). This finding is in contrast with a previous report that suggests no immediate difference in fibronectin expression upon YAP knockdown in cancer cells [57].

**Figure 6:**
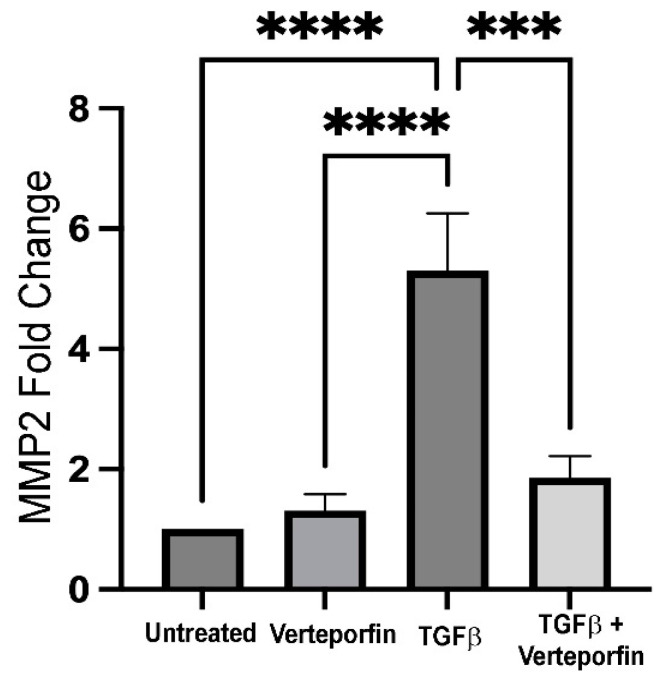
Expression of MMP2 upon YAP inhibition. cDNA reverse transcribed from RNA isolated from lens explants incubated with TGFβ in the presence or absence of verteporfin were amplified using PCR. GAPDH was used as a housekeeping gene control. Graph shows 5-fold increase in MMP2 expression in TGFβ-treated LECs when compared to untreated LECs (normalized with GAPDH) (ANOVA–Tukey multiple comparison test); **** *p* < 0.0001 untreated (vehicle-treated) or verteporfin-treated LECs vs. TGFβ-treated LECs). Inhibition of YAP signaling results in ~3.5-fold decrease in MMP2 expression when compared to TGFβ-treated LECs (*** *p* < 0.001 TGFβ-treated LECs vs. TGFβ + verteporfin-treated LECs). (*n* = 3 independent experiments, with each experiment consisting of at least 6 LECs per treatment group). Error bars indicate standard deviation.

During fibrosis, remodeling of ECM is an important process that facilitates cellular transformation and provides migratory capabilities to the transformed epithelial cells, and the master regulators of ECM remodeling are MMPs. We, and others, using in vivo and ex vivo model systems, have demonstrated the importance of both MMP2 and MMP9 in lens EMT and fibrosis [21,42,43,44,58,59]. For example, whole lenses and LECs from MMP9 mice demonstrated resistance against TGFβ-induced lenticular plaque formation and capsular wrinkling, respectively, and showed the absence of the expression of the key EMT marker, α-SMA [21]. In addition to MMP9, the inhibition of MMP2 by siRNA and neutralizing antibodies prevented a TGFβ-induced capsular contraction and morphological changes in LECs [42]. While there is no evidence of regulation of MMPs by YAP in the lens upon induction by TGFβ, knockdown of YAP using siRNA results in upregulation of MMP2 in mammary epithelial cancer cells [41]. In contrast, a recent report found positive changes in chromatin accessibility at genomic loci, resulting in upregulation of MMP2, MMP3, MMP10, MMP13, and MMP14 in connective tissue cells over-expressing constitutively active YAP [60]. In agreement with the later findings, we observed a significant downregulation of TGFβ-induced MMP2 expression in LECs upon YAP inhibition when compared to LECs treated with TGFβ alone (Figure 6). In addition to MMP2, we also observed a decrease, but not significant, in TGFβ-induced upregulation of MMP9 in LECs incubated with both TGFβ and verteporfin when compared to LECs treated with TGFβ alone (Figure S2). It is possible that the downregulation of MMP2 expression upon YAP inhibition is sufficient to prevent TGFβ-induced EMT. The differences in our observations compared to work from others [41] might be due to the system, i.e., primary epithelial cells vs. cancer cells, that was employed to study the function of YAP upon stimulation by TGFβ. It is also possible that the complete knockdown of the YAP gene vs. inhibition of the YAP protein might have different consequences in modulating downstream signaling through differential inhibition of its interaction with partner proteins.

Taken together, for the first time, we have shown the presence of nuclear YAP in human ASC capsules and an increase in YAP expression in mouse lenticular plaques upon upregulation of TGFβ in the lens. Our results demonstrate that inhibition of YAP prevents TGFβ-induced upregulation of fibronectin, α-SMA, and MMP2 in addition to blocking the delocalization of E-cadherin and β-catenin in LECs. Collectively, these observations suggest that YAP might be regulating TGFβ-mediated lens EMT at multiple levels: (1) upstream of TGFβ signaling through its regulation of fibronectin expression, (2) downstream of TGFβ signaling through modulation of TGFβ-induced delocalization of β-catenin, and (3) through regulating the expression of MMP2, one of the main matrix contraction proteins induced during TGFβ signaling. However, further studies are warranted to gain mechanistic insights into how YAP regulates the progression of EMT and to understand the dependence of canonical TGFβ signaling on YAP during TGFβ-induced EMT and lens fibrosis.

## 5. Conclusions

Our observations show the requirement of YAP signaling during TGFβ-induced lens EMT as one of the main causes of fibrotic cataracts including ASC and PCO. Our findings suggest that YAP could be tested as a potential therapeutic target against fibrotic cataracts in a clinical setting.

## Data Availability

The data presented in this study are available in this article.

## Supplementary Materials

The following supporting information can be downloaded at https://www.mdpi.com/article/10.3390/biom13121767/s1. Figure S1: Status of TAZ upon YAP inhibition. Figure S2: Expression of MMP9 upon YAP inhibition.
