# Supplementary material for: Understanding the Role of Yes-Associated Protein (YAP) Signaling in the Transformation of Lens Epithelial Cells (EMT) and Fibrosis
Source: Biomolecules. 2023 Dec 9;13(12):1767. doi: 10.3390/biom13121767 (PMC10741558; doi:10.3390/biom13121767)
Supplement: Supplementary file 1 [file biomolecules-13-01767-s001.zip › biomolecules-2716275-supplementary.pdf]

Supplementary Figures

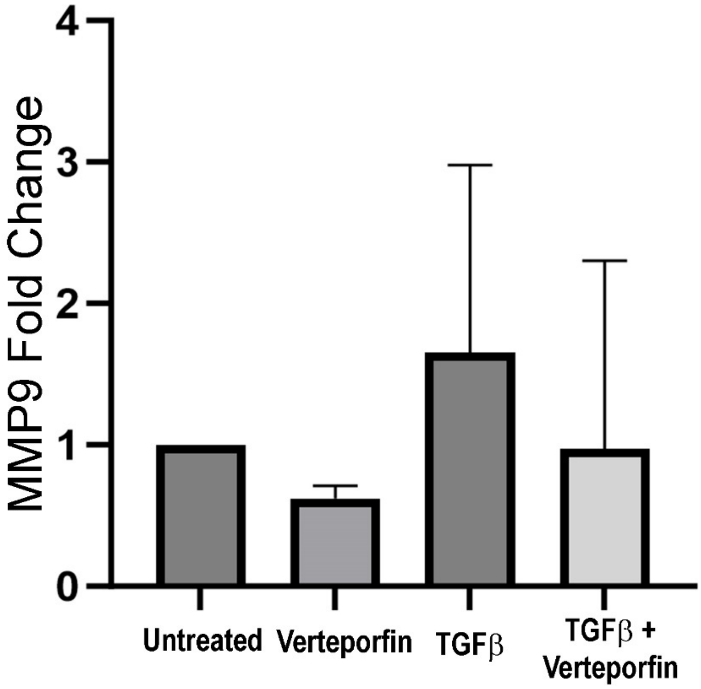

Figure S1 – Expression of MMP9 upon YAP inhibition. cDNA reverse transcribed from RNA isolated from lens explants incubated with TGFβ in the presence or absence of verteporfin were amplified using PCR. GAPDH was used as a housekeeping gene control. Graph shows 1.6-fold increase in MMP9 expression in TGFβ-treated LECs when compared to untreated LECs (normalized with GAPDH). Inhibition of YAP signaling results in ~1.7-fold decrease in MMP9 expression when compared to TGFβ-treated LECs. The results were non-significant. (ANOVA–Tukey multiple comparison test; n=3 independent experiments with each experiment consisting of at least 6 LECs per treatment groups). Error bars indicate standard deviation.

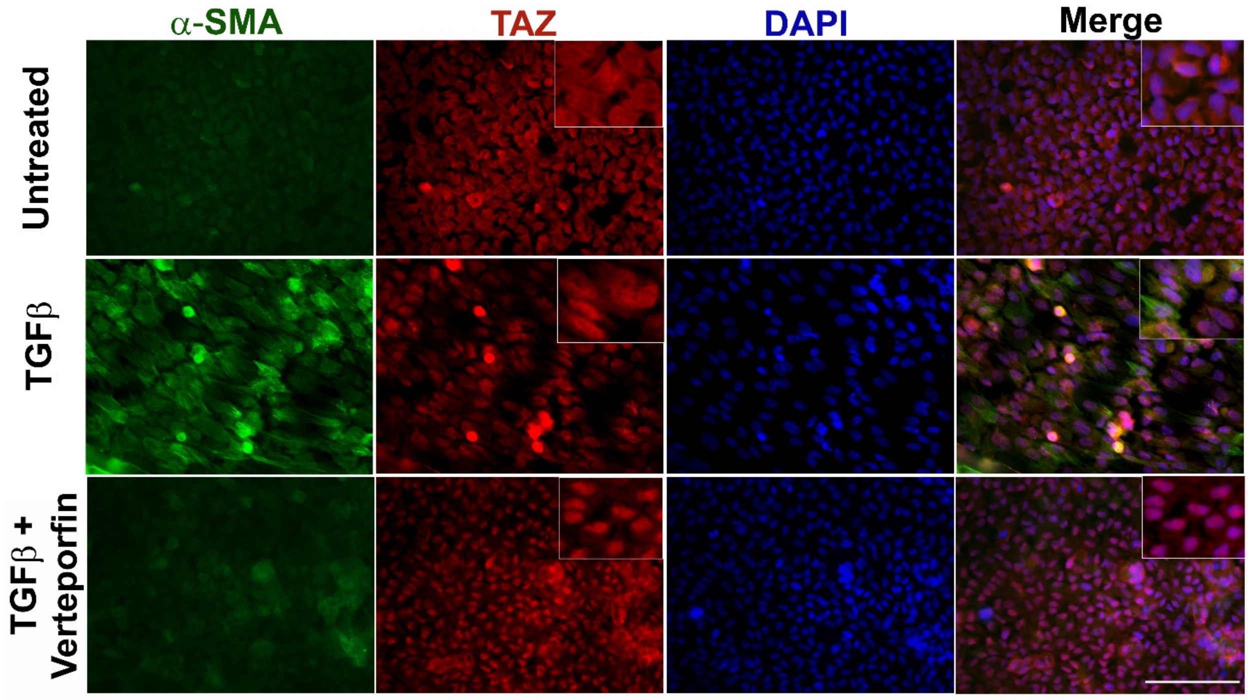

Figure S2 - Status of TAZ upon YAP inhibition. Rat LECs treated with TGFβ alone (6ng/mL) or along with verteporfin (100nM), were incubated for 48 hours (n=9 explants per treatment) and then fixed in PFA followed by staining for *α*-SMA (green) and TAZ (red). Incubation of rat LECs with TGFβ results in nuclear localization of TAZ. Inhibition of YAP by verteporfin was not able to prevent TGFβ-induced nuclear translocation of TAZ. Insets are cropped and magnified images of respective treatment groups that clearly shows the presence or absence of nuclear TAZ. Images were obtained using the 40x lens of Leica DM6 fluorescence microscope with scale bars set to 100 μm.
